# Role of complex energy and momentum in open cavity resonances

**DOI:** 10.1515/nanoph-2024-0623

**Published:** 2025-02-17

**Authors:** DongJun Kang, Eun Su Jeon, SeokJae Yoo

**Affiliations:** Department of Physics, 26718 Inha University, Incheon, Republic of Korea; Department of Physics, Program in Semiconductor and Device, Research and Education on Next-Generation Semiconductor Materials and Devices for Chiplet Technology, 26718 Inha University, Incheon, Republic of Korea

**Keywords:** conservation laws, energy, momentum, quasi-normal modes, open cavity, resonance

## Abstract

Complex power, also known as alternating current (AC) power, is a well-established concept in an electric circuit composed of resistive and reactive elements. On the other hand, the role of complex power in optics has been elusive. In this work, we reveal that the complex energy and momentum determine the resonance frequency and the decay rate of open cavity resonance, the so-called quasinormal modes (QNMs), respectively. We also demonstrate the role of the complex energy and momentum in typical open cavities analytically and numerically: the Fabry–Perot cavity, the surface plasmon polaritons (SPPs), the plasmonic nanorod, the nanosphere, and the dielectric supercavity.

## 1 Introduction

Light–matter interaction is mediated by the transfer of conserved quantities; for example, the momentum of light transports electromagnetic energy to excite an object, resulting in many optical phenomena such as optical force, optical torque, absorption, scattering, fluorescence, and circular dichroism [1], [2], [3]. Conserved quantities such as energy, and their mediator, i.e. momentum, are of fundamental importance in understanding light–matter interaction.

Energy conservation also plays an important role in the resonance characteristics of the optical cavity. The response of an optical system to arbitrary excitation is composed of its building blocks, namely normal modes, a free electromagnetic motion in the absence of the excitation. For isolated optical cavities that have no energy loss from radiation and Ohmic dissipation, the normal modes are defined by the energy stored by the cavity. In formal words, the electromagnetic wave equation for the isolated system can be understood as the Hermitian eigenvalue problem [4], [5]. Its eigenvalues, i.e. the resonance frequencies of the normal modes, are real values, while its eigenfunctions, i.e. the electromagnetic fields, are normalizable by the conserved energy.

Realistic optical cavities, however, are open cavities that have energy loss from radiation to free space in addition to Ohmic dissipation in lossy materials. Since energy is not conserved, the normal modes are not well-defined. Instead, solutions of the non-Hermitian eigenvalue problems of the open cavities exist, and they are called the quasinormal modes (QNMs) [6], [7], [8], [9], [10], [11]. Again, a set of QNMs describes the response of an open cavity to arbitrary excitation. Interestingly, QNM has a complex resonance frequency ${\tilde{\omega }}_{0}=\omega_{0}-i\gamma_{0}$ , whose real and imaginary part describe the real resonance frequency and the temporal decay rate of QNM, respectively.

Although energy is not conserved in QNMs, it is possible to write the energy conservation law taking loss into account. The energy conservation law also characterizes the decay rate, one of the fundamental measures of QNM. It has been suggested that the ratio of energy stored to the external transfer of momentum defines the decay rate (or the quality factor Q) of resonant cavities [6], [7]. However, there has been no complete picture of the relation between conserved quantities and the characteristics of open-cavity resonances.

On the other hand, it also has been known that electromagnetic fields have an infinite number of conserved quantities [12], but only a few of them have physical meanings, e.g. the optical chirality [13] and some of the Lipkin zilches [14]. One of the interesting conservation laws is the complex-valued energy/momentum. In the electrical circuit with the complex impedance $\tilde{Z}$ , the energy rate transferred to the electrical elements becomes the complex power, while its real (imaginary) part, i.e. the active (reactive) power, is related to the resistive (reactive) element [15]. However, in general electrodynamics, how the complex energy is defined and what carries the complex energy have been elusive, especially in terms of the electromagnetic fields, although some classic textbooks attempted to relate the complex impedance with the energy conservation law (i.e. the Poynting’s theorem) [16], [17].

In this work, we find the conservation law of the complex energy and its physical meanings in open cavity systems. The real energy/momentum gives the well-known energy balance; the real energy, the so-called active energy, is conserved, while it is transported by the active momentum. Its ratio defines the decay rate of the optical cavity. On the other hand, the imaginary energy/momentum, namely the reactive energy/momentum, also forms the conservation law, while they define the resonance frequency of the optical cavity. We also provide analytic and numerical examples of the optical cavity, e.g. the Fabry–Perot cavity, the surface plasmon polaritons, the gold nanorod, the gold nanosphere, and the dielectric supercavity to show the roles of the active/reactive energy/momentum in the open cavity resonance.

## 2 Conservation laws of open-cavity resonances

### 2.1 Complex energy conservation law

**Figure 1:**
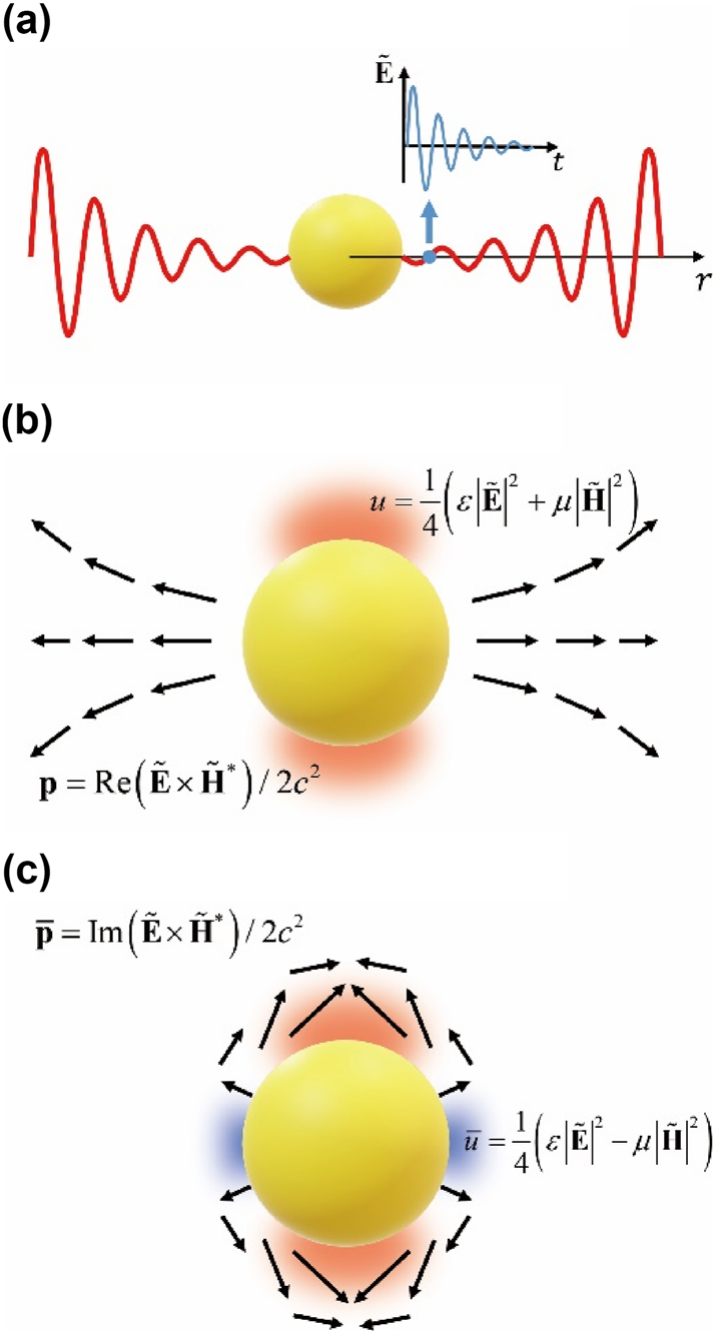
A schematic drawing of (a) the spatial amplification and the temporal decay of the quasinormal mode, (b) the active energy and momentum and (c) their reactive counterparts of the dipolar quasinormal mode of the plasmonic nanoparticle whose complex frequency is ${\tilde{\omega }}_{0}=\omega_{0}-i\gamma_{0}$ . In (b) & (c), the red and blue regions show the positive and negative regions of the active/reactive energy density (*u* and $\bar{u}$ ), respectively. The black arrows depict the active/reactive momentum density (**p** and $\bar{p}$ ). The active energy/momentum extends to the far field due to the radiation, defining decay rate *γ*_0_ of the nanoparticle. On the other hand, the reactive energy/momentum is strongly localized near the surface, defining the resonance frequency *ω*_0_. The numerically calculated profiles of the active/reactive energy/momentum near the nanoparticle are shown in Figure S1 (see Supplementary Information).

Resonators in the real world suffer from radiative and Ohmic energy loss. Once the lossy resonator, namely the open-cavity resonator, is excited by the external field, it decays over time with the finite quality factor *Q*, while oscillating with the characteristic resonance frequency *ω*_0_. To take into account the damped oscillation of the open-cavity resonance, the resonance frequency becomes the complex values, ${\tilde{\omega }}_{0}=\omega_{0}-i\gamma_{0}$ , whose real (*ω*_0_) and imaginary part (*γ*_0_ = *ω*_0_/2*Q*) describe the real resonance frequency and the temporal decay rate, respectively. Note that the tilde denotes the complex quantities throughout the paper. Formally, the temporally damped oscillation behaviors of the open-cavity resonance can be described by the concept of the quasinormal modes (QNMs), solutions of the non-Hermitian source-free electromagnetic wave equation with the complex-valued eigenfrequency. A direct result of the temporal decay of the QNM fields, i.e. $\exp\left(-i{\tilde{\omega }}_{0}t\right)=\exp\left(-i\omega_{0}t\right)\exp\left(-\gamma_{0}t\right)$ , is the spatial amplification of the field, i.e. $\exp\left(i{\tilde{k}}_{0}\cdot r\right)=\exp\left(ik_{0}\cdot r\right)\exp\left(\kappa_{0}\cdot r\right)$ with the complex momentum $\tilde{k}=k-i\kappa$ , as depicted in Figure 1(a). This temporal decay-spatial amplification makes QNMs stationery in spacetime.

One of the interesting, but physically vague conserved quantities is the complex momentum density of light, $\tilde{p}=p+i\bar{p}=\left(\tilde{E}\times {\tilde{H}}^{*}\right)/2c^{2}$ ; its real part corresponds to the well-known momentum density p=ReE~×H~*/2c2 that delivers the electromagnetic energy, but the physical role of its imaginary part, the so-called reactive momentum density [17], p¯=ImE~×H~*/2c2 is relatively unfamiliar in general electromagnetics. On the other hand, it has been recently reported that the imaginary part $\bar{p}$ has physical significance in the three specific cases: (i) $\bar{p}$ determines the electric-magnetic interaction component of the optical force on small particles with the electric and magnetic dipole moment [18], [19], [20], [21], [22], [23]. (ii) The diagonally polarized evanescent waves can deliver $\bar{p}$ and it is related to the transverse spin momentum density [19], [21], [22]. (iii) The azimuthally polarized beam can form the vortex of $\bar{p}$ [23].

Here, we reveal that a pair of two momentum densities defines the complex resonance frequency in open-cavity resonance. Suppose we have a single-mode open-cavity with a complex resonance frequency ${\tilde{\omega }}_{0}$ . We can show that the following pair of two continuity equations in the nondispersive lossless dielectric medium can be obtained directly by Maxwell’s equations for QNMs (see Supplementary Material for the derivation):

(1) c2∇⋅p+2Re−iω~0u=0,

(2) c2∇⋅p¯+2Im−iω~0u¯=0.

Here, the active and reactive energy densities are defined respectively by

(3) $$u=\frac{1}{4}\left(\varepsilon {\left|\tilde{E}\right|}^{2}+\mu {\left|\tilde{H}\right|}^{2}\right),$$

(4) $$\bar{u}=\frac{1}{4}\left(\varepsilon {\left|\tilde{E}\right|}^{2}-\mu {\left|\tilde{H}\right|}^{2}\right),$$

where $\left\{\tilde{E},\tilde{H}\right\}$ is a set of the electric and auxiliary magnetic fields. Eq. (1) corresponds to the energy conservation law (or the Poynting theorem) for QNMs, describing the transport of the momentum density **p** and the resulting changes in the energy density *u*. On the other hand, the physical meaning of Eq. (2), the imaginary part of the complex Poynting theorem, is elusive although there have been tries to interpret Eq. (2) using the lumped circuit concepts [16].

### 2.2 Physical meaning of complex energy and momentum

To find the physical meaning of Eq. (2) and the reactive energy/momentum, we can write its corresponding integral expression. Since Eqs. (1) and (2) are well defined in all spaces, we can convert them into the integral form for the arbitrary volume *τ* enclosed by the surface Σ in free space outside the open-cavity resonator. Eqs. (1) and (2) yield

(5) $$\gamma_{0}=\frac{c^{2}}{2}\frac{\underset{\Sigma }{\int }p\left(r\right)\cdot da}{\underset{\tau }{\int }u\left(r\right)dV}=\frac{c^{2}}{2}\frac{\nabla \cdot p\left(r\right)}{u\left(r\right)},$$

(6) $$\omega_{0}=-\frac{c^{2}}{2}\frac{\underset{\Sigma }{\int }\bar{p}\left(r\right)\cdot da}{\underset{\tau }{\int }\bar{u}\left(r\right)dV}=-\frac{c^{2}}{2}\frac{\nabla \cdot \bar{p}\left(r\right)}{\bar{u}\left(r\right)},$$

respectively. We emphasize that both integral and differential forms are valid. This implies the electromagnetic fields of QNM over the whole space include temporal information of the QNM. Again, Eq. (5) reads two ways: (i) the energy balance between the energy flux leaving through the closed surface Σ and the stored energy in the volume *τ* [6], [7] and (ii) the energy-momentum ratio. In the same way, we can understand Eq. (6) as (i) the balance between the reactive energy flux and the reactive energy or (ii) the reactive energy-reactive momentum ratio. Interestingly, the left-hand sides of Eqs. (5) and (6) are the temporal quantities, but the right-hand sides are the spatial quantities. This means that the electromagnetic fields of QNMs encode temporal information in every space.

To demonstrate physical meaning of complex energy and momentum, Figure 1 illustrates the active/reactive energy and momentum near the plasmonic nanoparticle at the dipolar QNM with the complex frequency ${\tilde{\omega }}_{0}=\omega_{0}-i\gamma_{0}$ . The dipolar character of the resonance radiates the energy *u* confined by the nanoparticle, and thus the momentum **p**, i.e. the energy carrier, survive outside the nanoparticle (Figure 1(b)). Therefore, their ratio defines the imaginary part of the complex frequency Imω~0=γ0 , i.e. the decay rate as shown in Eq. (5). It is straightforward to understand the role of the active energy/momentum in the open cavity resonance by Eq. (5). In a similar manner, we can understand that of the reactive energy/momentum using Eq. (6). The real part of the complex frequency Reω~0=ω0 , i.e. the resonance frequency, and its relation with $\bar{u}$ and $\bar{p}$ explain how the electromagnetic field is strongly confined to the open cavity. In Figure 1(c), the reactive momentum $\bar{p}$ do not survive far from the nanoparticle, but it is strongly localized near the surface. The reactive energy $\bar{u}$ also explains where $\bar{p}$ directs and how it is localized in the near-field zone. Vanishing $\bar{u}$ and $\bar{p}$ in the far-field zone of the nanostructure can be understood by the definition of the reactive energy $\bar{u}$ ; $\bar{u}$ is defined by the difference in the electric and magnetic parts of the energy density as shown in Eq. (4). In the far-field zone away from the nanostructure, the electromagnetic field becomes the plane wave-like. The plane wave stores the energy in the electric and the magnetic parts at the same amount. Therefore, $\bar{u}$ is always zero in the far-field, while it is meaningful only in the near-field. In the next section, we demonstrate physical meaning of complex energy and momentum using the actual examples of the open cavity nanostructures.

## 3 Complex energy and momentum in the open cavity nanostructures

### 3.1 One-dimensional (1D) Fabry–Perot cavity

**Figure 2:**
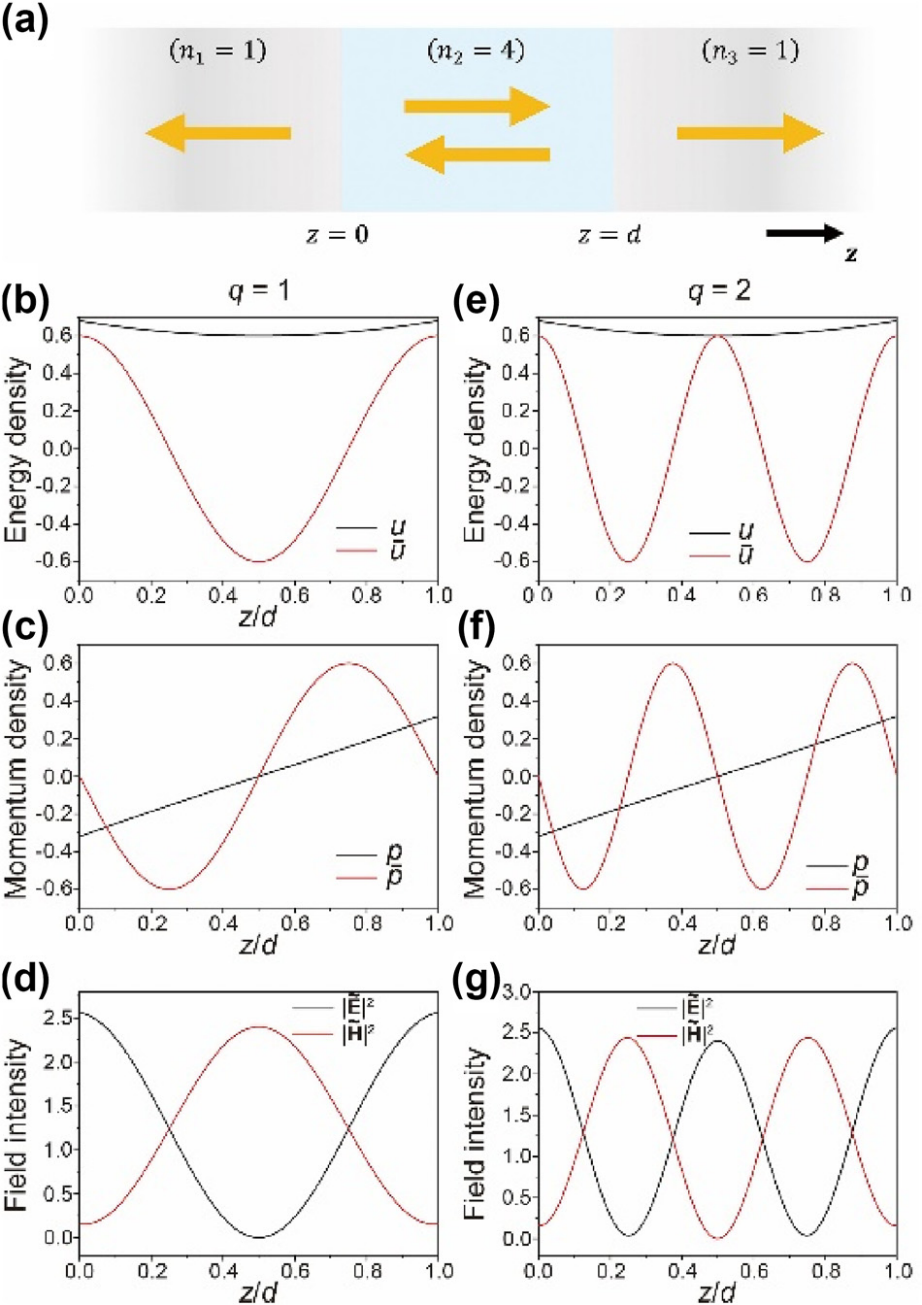
The active/reactive energy/momentum of (a) the 1D dielectric Fabry–Perot cavity (*n*_2_ = 4) in air (*n*_1_ = *n*_3_ = 1). Left and right columns correspond to the resonance order of *q* = 1 and 2, respectively. The yellow arrows indicate the directions of the electric fields, Eq. (7). (b, e) The active (black) and reactive (red) energy densities. (c, f) The active (black) and reactive (red) momentum densities. (d, g) The electric (black) and magnetic (red) field intensities. The active/reactive energy densities and momentum densities are normalized by the factors $\varepsilon_{2}{\left|{\tilde{E}}_{2}\right|}^{2}$ and $n_{2}{\left|{\tilde{E}}_{2}\right|}^{2}/c^{2}\eta_{0}$ , respectively.

The simplest example in optics is the 1D Fabry–Perot cavity (Figure 2(a)). Each region has the purely real-valued refractive index *n*_*i*_ (*i* = 1, 2, and 3) for sake of the simplicity. Length of the cavity, i.e. the medium 2, is *d*. The electric fields are written as

(7) $$\begin{array}{cc} \tilde{E}\left(z\right) & ={\tilde{E}}_{1}e^{-i{\tilde{k}}_{1}z}e^{-i{\tilde{\omega }}_{0}t}\hat{x}\;\left(z\leq 0\right)\\ & ={\tilde{E}}_{2}\left(r_{21}{\text{e}}^{\text{i}{\tilde{k}}_{2}z}+e^{-i{\tilde{k}}_{2}z}\right)e^{-i{\tilde{\omega }}_{0}t}\hat{x}\;\left(0\leq z\leq d\right)\\ & ={\tilde{E}}_{3}{\text{e}}^{\text{i}{\tilde{k}}_{3}\left(z-d\right)}e^{-i{\tilde{\omega }}_{0}t}\hat{x}\;\left(z\geq d\right) \end{array},$$

where ${\tilde{E}}_{i}$ is the field amplitude in the *i*-th medium. ${\tilde{k}}_{i}=n_{i}{\tilde{k}}_{0}$ and ${\tilde{k}}_{0}$ are the wavenumber in the *i*-th medium and the free space, respectively. The Fresnel equation provides $r_{ij}=\left(n_{i}-n_{j}\right)/\left(n_{i}+n_{j}\right)$ , i.e. the reflection coefficient at the interface between the medium *i* and *j*. The corresponding magnetic fields can be obtained by one of the Maxwell’s equations, $\tilde{H}=\left(\nabla \times E\right)/i\tilde{\omega }\mu_{0}$ . The condition for the bound solution, $r_{12}r_{23}e^{2in_{2}{\tilde{k}}_{0}d}+1=0$ , gives the resonance condition of the Fabry–Perot cavity,

(8) $${\tilde{k}}_{0}=q\frac{\pi }{n_{2}d}-i\frac{1}{2n_{2}d}\ln\left(\frac{1}{r_{21}r_{23}}\right)\;\left(q=1,2,3,\ldots \right).$$

Here, the complex frequency is given by the dispersion of light, ${\tilde{\omega }}_{0}=c{\tilde{k}}_{0}$ . We can find that finite reflectivity gives the imaginary part of ${\tilde{k}}_{0}$ and ${\tilde{\omega }}_{0}$ although the material loss is absent, i.e. the vanishing imaginary part of the refractive index. From Eq. (7), we can obtain the active and reactive energy densities inside the cavity $\left(0\leq z\leq d\right)$ ,

(9) u=12ε2E~22r212e−2Imk~2z+e+2Imk~2ze−2γ0t,

(10) u¯=ε2E~22r21⁡cos2Rek~2ze−2γ0t,

respectively. The active and reactive momentum densities inside the cavity are given by

(11) p=n22c2η0E~22r212e−2Imk~2z−e+2Imk~2ze−2γ0tz^,

(12) p¯=−n2c2η0E~22r21⁡sin2Rek~2ze−2γ0tz^,

respectively. We can find that Eqs. (9)–(12) satisfy Eqs. (5) and (6). On the other hand, the reactive energy density ( $\bar{u}$ ) and momentum density ( $\bar{p}$ ) vanish outside the cavity (*z* ≤ 0 or *z* ≥ *d*), and thus they cannot provide *ω*_0_ by Eq. (6).

Figure 2(b)–(g) show the active/reactive energy density and the momentum density inside the cavity $\left(0\leq z\leq d\right)$ at the two lowest orders (*q* = 1 and 2). In Figure 2(a), cavity is composed of high-index material (*n*_2_ = 4), while two semi-infinite half spaces are filled with air (*n*_1_ = *n*_3_ = 1).

Since the definition of *u*, Eq. (3), guarantees its positive-definiteness, the energy density *u* is positive over the whole cavity space (the black line in Figure 2(b)). On the other hand, the reactive energy density $\bar{u}$ (the red line in Figure 2(b)) is positive near the walls (*z*/*d* = 0 and 1) and negative near the cavity center (*z*/*d* = 0.5) at the lowest order (*q* = 1). Positive (negative) reactive energy density $\bar{u}$ means dominance of the electric field energy density $u_{E}=\varepsilon {\left|\tilde{E}\right|}^{2}/4$ (the black line in Figure 2(d)) is larger (smaller) than the magnetic field energy density $u_{M}=\mu {\left|\tilde{H}\right|}^{2}/4$ (the red line in Figure 2(d)). On the other hand, the electromagnetic field outside the cavity diverges approaching to the infinity (*z* → ±∞) (see Supplementary Information), while the reactive energy and the divergence of reactive momentum vanish ( $\bar{u}$ = 0 and $\nabla \cdot \bar{p}$ = 0). We can conclude that $\bar{u}$ and $\nabla \cdot \bar{p}$ reflect the confinement characteristic of the electromagnetic fields because unconfined, but propagating waves outside the cavity do not deliver the reactive quantities.

In Figure 2(c), the momentum density **p** points to the outside from the cavity center (i.e. **p** = 0 at *z*/*d* = 0.5) because QNM is a bound mode that loses its energy by the radiation to the outside (*z* → ±∞). Also **p** shows linear behavior at both *q* = 1 and 2 because the exponential growth and decay terms can be expanded to the linear function (i.e. *e* ^*x*^ ≈ 1 + *x*), making Eq. (11) linear. In contrast, the reactive momentum density $\bar{p}$ shows sinusoidal behavior (Figure 2(c) and (f)), while it vanishes at the walls (*z*/*d* = 0 and 1) and outside (*z*/*d* < 0 and >1). Also, the reactive momentum density $\bar{p}$ and the reactive energy density $\bar{u}$ are independent from the cavity loss ( Imk~2=n2Imk~0 in Eq. (8)), but they are determined solely by the resonance characteristics *q* (the order) and *d* (the cavity length) by the term Rek~2=qπ/d . This implies the reactive momentum density reflects the confinement of the electromagnetic fields inside the cavity.

To sum up this section, the active energy and momentum densities are well-defined for all electromagnetic fields, but the reactive energy and the divergence of the reactive momentum do not vanish only for the confined fields, as shown in the 1D Fabry–Perot cavity. This can also be understood by Eqs. (5) and (6). In Eq. (5), the decay rate *γ*_0_ is solely determined by the active quantities; it is always determined by the ratio of the energy flux to the outside to the stored energy density. However, in Eq. (6), the reactive momentum and energy determines the resonance frequency *ω*_0_; the resonance occurs only when the energy is confined to the finite object. Therefore, the unconfined fields cannot have the reactive momentum flux and energy.

### 3.2 Surface plasmon polaritons (SPPs) at the metal/dielectric interface

We revisit SPPs at the metal/dielectric interface using the QNM formalism that has the complex frequency ${\tilde{\omega }}_{0}$ and the complex wave vectors ${\tilde{k}}_{0}$ . The semi-infinite half space (*z* > 0) is filled with the medium 1, dielectric with the permittivity *ɛ*_1_, while the opposite half-space (*z* < 0) is filled with the medium 2, metal with the complex permittivity ${\tilde{\varepsilon }}_{2}$ . The *x*-axis at the interface *z* = 0 defines the SPP propagation direction. The *p*-polarized electric field in the *i*-th medium (*i* = 1 and 2 for the dielectric and the metal, respectively) is given by

(13) $${\tilde{E}}_{i}={\tilde{E}}_{i,x}\left(\begin{array}{c} 1\\ 0\\ -{\tilde{k}}_{x}/{\tilde{k}}_{i,z} \end{array}\right)e^{i\left({\tilde{k}}_{x}x+{\tilde{k}}_{i,z}z-{\tilde{\omega }}_{0}t\right)},$$

satisfying $\nabla \cdot {\tilde{E}}_{i}=0$ . By the bound solution condition $\varepsilon_{1}{\tilde{k}}_{2,z}-{\tilde{\varepsilon }}_{2}{\tilde{k}}_{1,z}=0$ , the wave numbers are given by

(14) $${\tilde{k}}_{x}^{2}=\frac{1}{\varepsilon_{0}}\frac{\varepsilon_{1}{\tilde{\varepsilon }}_{2}}{\varepsilon_{1}+{\tilde{\varepsilon }}_{2}}{\tilde{k}}_{0}^{2},$$

(15) $${\tilde{k}}_{i,z}^{2}=\frac{1}{\varepsilon_{0}}\frac{\varepsilon_{i}^{2}}{\varepsilon_{1}+\varepsilon_{2}}{\tilde{k}}_{0}^{2},$$

while they are the same as the usual SPPs as follows [24], but all of them become complex values in the QNM formalism, while ${\tilde{k}}_{0}$ is linked to the complex resonance frequency by the relation ${\tilde{k}}_{0}^{2}={\tilde{\omega }}_{0}^{2}/c^{2}$ . The active energy density, the active momentum density, and the reactive momentum density in the nondispersive dielectric medium 1 are given by

**Figure 3:**
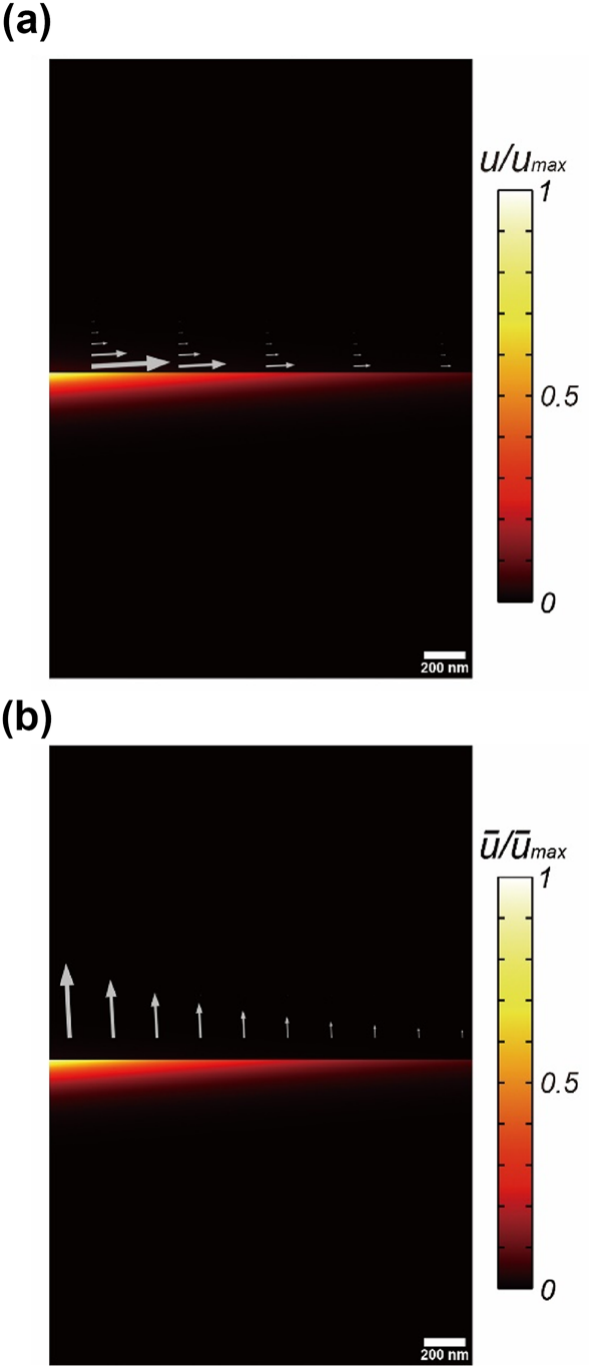
The active/reactive energy/momentum of the SPP QNM at the air-gold interface. (a) The active energy (color) and momentum densities (gray arrows). (b) The reactive energy (color) and momentum densities (gray arrows).

(16) u=ε12k~022ε0k~1,z2E~1,x2e−2Imk~xx+Imk~1,zze−2γ0t,

(17) p=12c2η0ε1ε0Rek~0*k~x/k~1,z20Rek~0*k~1,z/k~1,z2E~1,x2×e−2Imk~xx+Imk~1,zze−2γ0t,

(18) p¯=12c2η0ε1ε0Imk~0*k~x/k~1,z20Imk~0*k~1,z/k~1,z2E~1,x2×e−2Imk~xx+Imk~j,zze−2γ0t,

respectively. We can find that Eqs. (16) and (17) satisfy Eq. (5). In the SPP QNM, the reactive energy and the divergence of the reactive momentum vanish ( $\bar{u}$ = 0 and $\nabla \cdot \bar{p}$ = 0), making Eq. (6) indeterminate. Figure 3 shows the active/reactive energy/momentum density at the air-gold interface. To plot Figure 3, we use the electromagnetic fields of the SPP QNM, and The complex permittivity of gold at the SPP frequency is taken as ${\tilde{\varepsilon }}_{2}$ = −11.8 + 1.15*i*. The complex SPP frequency is given by ${\tilde{\omega }}_{0}/2\pi$ = (466.8 + 38.32*i*) THz.

The real SPPs (not in the QNM formalism) requires *ɛ*_1_ + *ɛ*_2_ < 0 and *ɛ*_1_ *ɛ*_2_ < 0 to make the plasmon momentum ${\tilde{k}}_{x}$ and the decay wavenumber ${\tilde{k}}_{i,z}$ pure real and pure imaginary values by Eqs. (14) and (15), respectively, if we neglect metallic loss (i.e. ${\tilde{\varepsilon }}_{2}=\varepsilon_{2}$ by Imε~2=0 ). The purely imaginary ${\tilde{k}}_{i,z}$ makes the direction of the momentum density **p**, Eq. (17), the *x*-direction along the surface (i.e. *p*_*x*_ ≠ 0 and *p*_*z*_ = 0). Contrarily, SPPs in the QNM formalism, the momentum density component normal to the surface *p*_*z*_ does not vanish, and thus the SPP QNM deliver some energy to the normal direction (the ±*z*-direction) as shown in Figure 3(a).

One of important feature in the SPP QNM is the vanishing reactive energy density and the divergence of the momentum density ( $\bar{u}$ = 0 and $\nabla \cdot \bar{p}$ = 0). This can be understood by the fact that the SPP QNM is not fully confined because it is bound to the surface (*z* = 0), but it propagates along the surface (i.e. the *x*-axis). This unbound character of the SPP QNM results in $\bar{u}$ = 0 and $\nabla \cdot \bar{p}$ = 0. The same was also shown in the 1D Fabry–Perot cavity in the previous section; outside the cavity, there are the unbounded, but propagating waves to the infinity, and they have $\bar{u}$ = 0 and $\nabla \cdot \bar{p}$ = 0. Note that the Fresnel equation for the SPPs have been derived analytically [25]. It can be shown that the reactive quantities start to appear when the SPPs are confined laterally on the interface to form the Fabry–Perot cavity as in the previous section.

To make the QNM of the metal structure have the reactive momentum and energy, the SPP should be confined by the optical cavity, resulting in the localized surface plasmon resonance (LSPR). For example, gold nanorods and nanospheres can localize SPPs within the finite structures and their very vicinity. In the next section, we numerically demonstrate that their reactive quantities do not vanish, but they survive near the structure surface.

### 3.3 Gold nanorod

**Figure 4:**
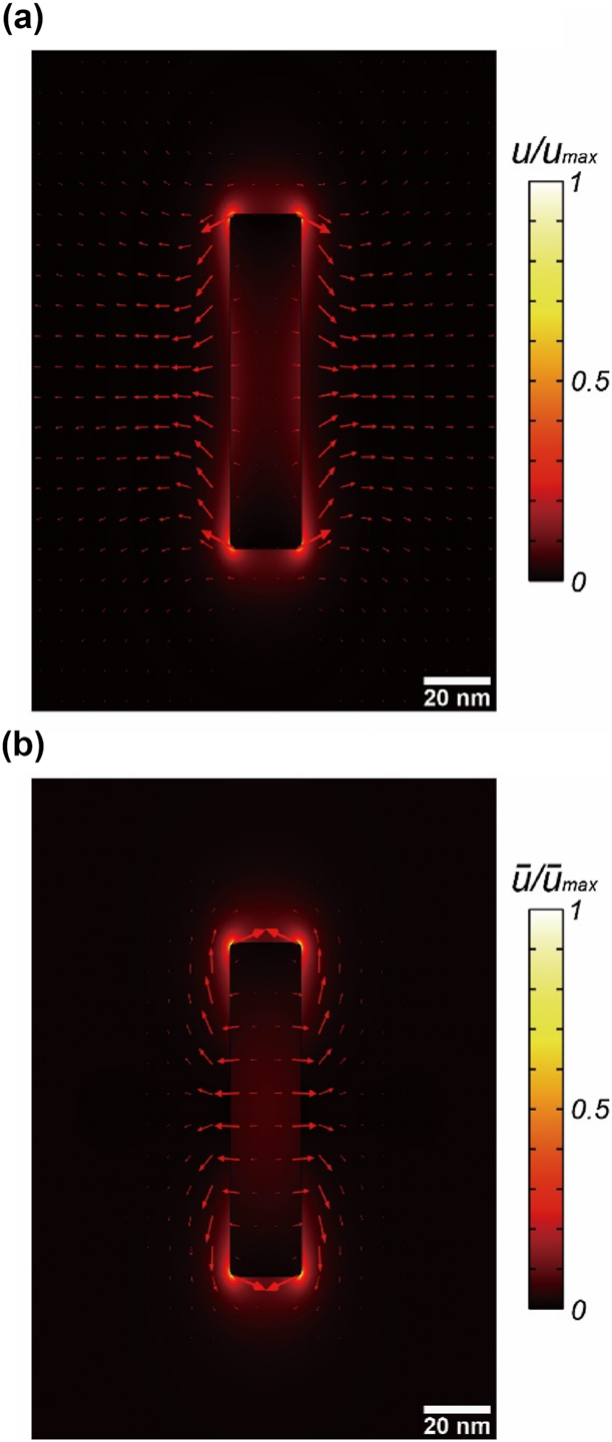
The active/reactive energy/momentum of the lowest QNM resonance of the cylindrical-shaped gold nanorod (the radius of 15 nm and the length of 100 nm) in air. (a) The active energy (color) and momentum densities (red arrows). (b) The reactive energy (color) and momentum densities (red arrows).

We have analysed the open cavity systems using the analytic expressions of the QNMs in the previous sections. Numerical techniques allow us to analyse the optical cavities that do not have analytic solutions in the closed form. Here, we calculate the QNM of a gold nanorod and its active/reactive energy and momentum in its vicinity in Figure 4. QNM was calculated by the MAN (Modal Analysis of Nanoresonators) package implemented by COMSOL Multiphysics [26]. The gold nanorod has a cylindrical shape with the length of 100 nm and the radius of 15 nm. The permittivity of gold was modelled by the Drude–Lorentz model [26]. MAN yields the dipolar resonance of the gold nanorod at the complex resonance frequency ${\tilde{\omega }}_{0}/2\pi$ = (318.1 + 11.66*i*) THz. The corresponding resonance wavelength and the quality factor are given by *λ*_0_ = 942.5 nm and *Q* = 27.28, respectively.

In Figure 4(a) and (b), the color profile shows the active energy density *u* (the reactive energy density $\bar{u}$ ), while the arrows show the active momentum density **p** (the reactive momentum density $\bar{p}$ ). Both the active and reactive energy densities are concentrated near the gold nanorod surface because of the localized surface plasmon resonance (LSPR). However, the gold nanorod loses its energy by the radiation, and thus the active momentum density **p** extends to the far-field in Figure 4(a). In contrast, the reactive momentum density $\bar{p}$ is strongly localized only near the surface. This is consistent with the 1D Fabry–Perot cavity and SPP in the previous section. The reactive momentum and energy reflect the confinement character of the optical system, and thus they vanish outside the cavity. On the other hand, $\bar{p}$ can survive in the very vicinity of the gold surface, but it rapidly vanishes outside the nanorod (Figure 4(b)); the gold nanorod does not have physical walls of the cavity. Therefore, the free space near the nanorod is also part of the cavity.

In addition to the plasmonic structures we have studied in Figures 1, 3, 4 and S1, we also numerically analyze the active/reactive energy/momentum of a dielectric open-cavity that can have the high *Q* factor [27], [28], [29], [30]. In the Supplementary Material, we calculate the QNM of a high-index dielectric nanodisk. One of its QNMs (Figure S2) corresponds to the so-called supercavity mode at the high-Q bound states in the continuum [28]. We find Eqs. (5) and (6) also works well for the dielectric supercavity.

We also emphasize that the temporal information of the QNM, *ω*_0_ and *γ*_0_, can be obtained by the spatial information of $\tilde{E}\left(r\right)$ and $\tilde{H}\left(r\right)$ by Eqs. (5) and (6). These relations are valid everywhere in the simulation domain, except for the singular point of Eqs. (5) and (6), i.e. the points where the active or reactive energy density vanish. It is also noteworthy that Eqs. (5) and (6) are valid in the usual eigenfrequency calculation in COMSOL Multiphysics even though the MAN package is not used.

## 4 Conclusions

We reveal that the complex energy/momentum provide complete description of the open cavity resonance, i.e. QNM; the active energy/momentum describes how the optical cavity stores and loses the actual (real-valued) energy. It is related to the decay rate of the cavity (Eq. (5)). The reactive energy/momentum describes how the optical cavity confines the electromagnetic fields inside and in the vicinity of the cavity structure. It defines the resonance frequency (Eq. (6)). This finding can expand our understanding of the open cavity resonance, and it can be helpful to design the resonant optical cavity.

Before concluding, the following three points are noteworthy; (i) Eqs. (5) and (6) can be extended to the lossy medium and the dispersive medium (see Supplementary Information for details). These expressions are useful when the energy and the momentum densities are evaluated inside the nanostructures. (ii) We are also able to derive the active/reactive pair of the optical helicity and its carrier, i.e. the spin angular momentum of light (see Supplementary Information for details). Their ratios also give the similar expression to Eqs. (5) and (6). This can be useful to analyze QNMs of the chiral nanostructure. (iii) It is also noteworthy that the complex resonance frequency, Eqs. (5) and (6), is normalized by the field intensities, ${\left|\tilde{E}\right|}^{2}=\tilde{E}\cdot {\tilde{E}}^{*}$ and ${\left|\tilde{H}\right|}^{2}=\tilde{H}\cdot {\tilde{H}}^{*}$ . In QNMs, the fields $\tilde{F}\in \left\{\tilde{E},\tilde{H}\right\}$ are normalized by $\tilde{F}\cdot \tilde{F}$ rather than ${\left|\tilde{F}\right|}^{2}=\tilde{F}\cdot {\tilde{F}}^{*}$ because of the broken energy conservation in the open cavities [7], [8], [31], [32]. The QNM formalism guarantees the normalization of the open cavities, but it is hard to interpret quantities physically. However, as shown in Eqs. (5) and (6), it is possible to define physical quantities using physically meaningful expressions based on ${\left|\tilde{F}\right|}^{2}$ in the QNM formalism.

## Supplementary Material

Supplementary Material Details
