# Supplementary material for: Role of complex energy and momentum in open cavity resonances
Source: Nanophotonics. 2025 Feb 17;14(8):1231–8. doi: 10.1515/nanoph-2024-0623 (PMC12019941; doi:10.1515/nanoph-2024-0623)
Supplement: Supplementary file 1 — Supplementary Material Details [file j_nanoph-2024-0623_suppl_001.pdf]

Role of complex energy and momentum in open cavity resonances

DongJun Kang, Eun Su Jeon, and SeokJae Yoo

1. Active/reactive energy/momentum density of the gold nanosphere

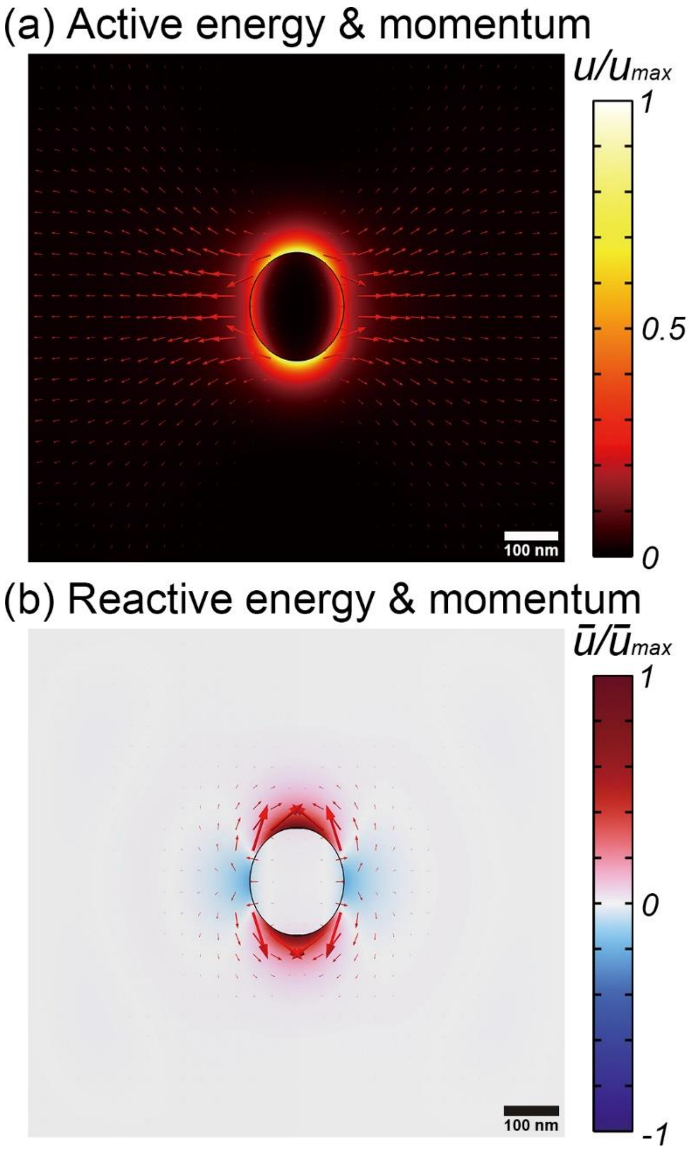

Fig. S1: The active/reactive energy/momentum density of the gold nanosphere. (a) The active energy density (color) and the active momentum density (red arrows). (b) The reactive energy density (color) and the reactive momentum density (red arrows). In Fig. S1b, red and blue regions show the positive and negative reactive energy densities, respectively.

2. Active/reactive energy/momentum density of the Silicon nanodisk

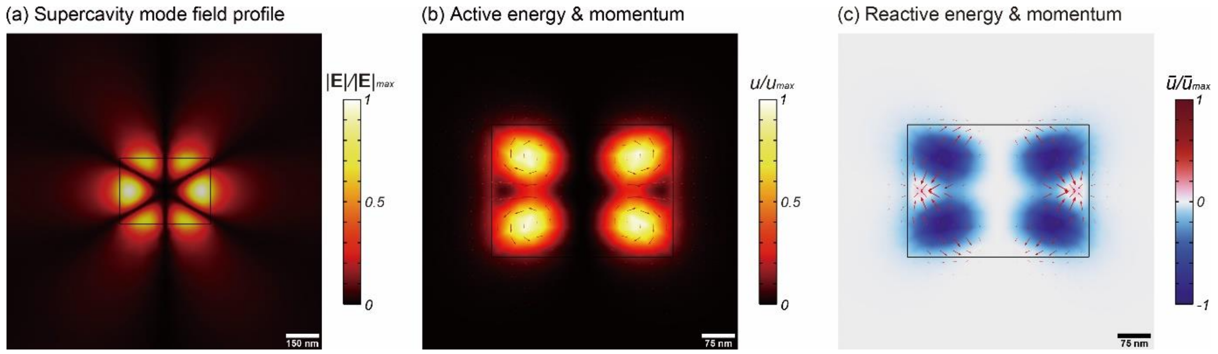

Fig. S2: The active/reactive energy/momentum density of the Si nanodisk. (a) The normalized electric field profile. (b)The active energy density (color) and the active momentum density (red arrows). (c) The reactive energy density (color) and the reactive momentum density (red arrows). In Fig. S1b, red and blue regions show the positive and negative reactive energy densities, respectively.

3. Energy conservation in quasinormal modes (QNMs)

Quasinormal modes (QNMs) are solution of the non-Hermitian eigenvalue problem, and they have the complex resonance frequency $\tilde{\omega}_0 = \omega_0 - i\gamma_0$. The imaginary part of the resonance frequency corresponds to the QNM decay rate, and it is also related to the quality factor *Q* by the relation $\gamma_0 = \omega_0 / Q$. Suppose $\{\tilde{\mathbf{E}}, \tilde{\mathbf{H}}\}$ is a set of the electric field and the auxiliary magnetic field, while $\{\tilde{\mathbf{D}}, \tilde{\mathbf{B}}\}$ is a set of the displacement field and the magnetic field. $\{\tilde{\mathbf{D}}, \tilde{\mathbf{B}}\}$ contains material information and it is linked to $\{\tilde{\mathbf{E}}, \tilde{\mathbf{H}}\}$ by the electromagnetic constitutive relation. QNM fields satisfy the Maxwell's equations,

$$\nabla \times \tilde{\mathbf{E}} = i\tilde{\omega}_0 \tilde{\mathbf{B}}, \quad \text{(S1)}$$

$$\nabla \times \tilde{\mathbf{H}} = -i\tilde{\omega}_0 \tilde{\mathbf{D}}, \quad \text{(S2)}$$

as well as their conjugated equations,

$$\nabla \times \tilde{\mathbf{E}}^* = -i\tilde{\omega}_0^* \tilde{\mathbf{B}}^*, \quad \text{(S3)}$$

$$\nabla \times \tilde{\mathbf{H}}^* = i\tilde{\omega}_0^* \tilde{\mathbf{D}}^*, \quad \text{(S4)}$$

Using Maxwell's equations, Eqs. (S1)-(S4), we can simplify the divergence of $\left(\tilde{\mathbf{E}} \times \tilde{\mathbf{H}}^*\right)$ as follows:

$$\nabla \cdot \left(\tilde{\mathbf{E}} \times \tilde{\mathbf{H}}^*\right) = -i\left(\tilde{\omega}_0^* \tilde{\mathbf{E}} \cdot \tilde{\mathbf{D}}^* - \tilde{\omega}_0 \tilde{\mathbf{H}}^* \cdot \tilde{\mathbf{B}}\right). \quad \text{(S5)}$$

The real and imaginary part of Eq. (S5) are explicitly written in the forms of Eqs. (1) and (2) in the main text.

1) Nondispersive and lossless linear dielectrics

If the medium is linear dielectric without dispersion and loss, we can write $\tilde{\mathbf{D}} = \varepsilon\tilde{\mathbf{E}}$ and $\tilde{\mathbf{B}} = \mu\tilde{\mathbf{H}}$ where $\varepsilon = \varepsilon_r\varepsilon_0$ and $\mu = \mu_r\mu_0$ are purely real values. Then, Eq. (S5) becomes $\nabla\cdot\left(\tilde{\mathbf{E}}\times\tilde{\mathbf{H}}^*\right) = -i\left(\tilde{\omega}_0^*\varepsilon\left|\tilde{\mathbf{E}}\right|^2 - \tilde{\omega}_0\mu\left|\tilde{\mathbf{H}}\right|^2\right)$. Taking the real and imaginary parts provides

$$\nabla\cdot\mathrm{Re}\left(\tilde{\mathbf{E}}\times\tilde{\mathbf{H}}^*\right) = \gamma_0\left(\varepsilon\left|\tilde{\mathbf{E}}\right|^2 + \mu\left|\tilde{\mathbf{H}}\right|^2\right), \tag{S6}$$

$$\nabla\cdot\mathrm{Im}\left(\tilde{\mathbf{E}}\times\tilde{\mathbf{H}}^*\right) = -\omega_0\left(\varepsilon\left|\tilde{\mathbf{E}}\right|^2 - \mu\left|\tilde{\mathbf{H}}\right|^2\right). \tag{S7}$$

Eq. (S6) and (S7) provide Eqs. (1) and (2) in the main text.

2) Nondispersive but lossy linear dielectrics

In this case, we can write $\tilde{\mathbf{D}} = \tilde{\varepsilon}\tilde{\mathbf{E}}$ and $\tilde{\mathbf{B}} = \tilde{\mu}\tilde{\mathbf{H}}$ where $\tilde{\varepsilon} = \tilde{\varepsilon}_r\varepsilon_0$ and $\tilde{\mu} = \tilde{\mu}_r\mu_0$ are complex values, but they are not functions of frequency. Then Eq. (S5) becomes $\nabla\cdot\left(\tilde{\mathbf{E}}\times\tilde{\mathbf{H}}^*\right) = -i\left(\tilde{\omega}_0^*\tilde{\varepsilon}^*\left|\tilde{\mathbf{E}}\right|^2 - \tilde{\omega}_0\tilde{\mu}\left|\tilde{\mathbf{H}}\right|^2\right)$. Taking the real and imaginary parts provides

$$\nabla\cdot\mathrm{Re}\left(\tilde{\mathbf{E}}\times\tilde{\mathbf{H}}^*\right) = -\mathrm{Im}\left(\tilde{\omega}_0\tilde{\varepsilon}\right)\left|\tilde{\mathbf{E}}\right|^2 - \mathrm{Im}\left(\tilde{\omega}_0\tilde{\mu}\right)\left|\tilde{\mathbf{H}}\right|^2, \tag{S8}$$

$$\nabla\cdot\mathrm{Im}\left(\tilde{\mathbf{E}}\times\tilde{\mathbf{H}}^*\right) = -\mathrm{Re}\left(\tilde{\omega}_0\tilde{\varepsilon}\right)\left|\tilde{\mathbf{E}}\right|^2 + \mathrm{Re}\left(\tilde{\omega}_0\tilde{\mu}\right)\left|\tilde{\mathbf{H}}\right|^2. \tag{S9}$$

By representing $\tilde{\varepsilon} = \varepsilon_1 + i\varepsilon_2$ and $\tilde{\mu} = \mu_1 + i\mu_2$, Eq. (S8) and (S9) becomes

$$\nabla\cdot\mathrm{Re}\left(\tilde{\mathbf{E}}\times\tilde{\mathbf{H}}^*\right) = -\omega_0\left(\varepsilon_2\left|\tilde{\mathbf{E}}\right|^2 + \mu_2\left|\tilde{\mathbf{H}}\right|^2\right) - \gamma_0\left(\varepsilon_1\left|\tilde{\mathbf{E}}\right|^2 + \mu_1\left|\tilde{\mathbf{H}}\right|^2\right), \tag{S10}$$

$$\nabla\cdot\mathrm{Im}\left(\tilde{\mathbf{E}}\times\tilde{\mathbf{H}}^*\right) = -\omega_0\left(\varepsilon_1\left|\tilde{\mathbf{E}}\right|^2 - \mu_1\left|\tilde{\mathbf{H}}\right|^2\right) + \gamma_0\left(\varepsilon_2\left|\tilde{\mathbf{E}}\right|^2 - \mu_2\left|\tilde{\mathbf{H}}\right|^2\right). \tag{S11}$$

3) Dispersive linear dielectrics

In the dispersive linear dielectrics, $\tilde{\mathbf{D}} = \tilde{\varepsilon}(\omega)\tilde{\mathbf{E}}$ and $\tilde{\mathbf{B}} = \tilde{\mu}(\omega)\tilde{\mathbf{H}}$ where $\tilde{\varepsilon}(\omega) = \tilde{\varepsilon}_r(\omega)\varepsilon_0$ and $\tilde{\mu}(\omega) = \tilde{\mu}_r(\omega)\mu_0$ are complex values. Fields in frequency domain are coming from Fourier transformations of time domain fields [1]

$$\tilde{\mathbf{E}}(\mathbf{r},t) = \int_{-\infty}^{\infty} d\tilde{\omega}\,\tilde{\mathbf{E}}(\mathbf{r},\tilde{\omega})e^{-i\tilde{\omega}t}, \tag{S12}$$

$$\tilde{\mathbf{D}}(\mathbf{r},t) = \int_{-\infty}^{\infty} d\tilde{\omega}\,\tilde{\mathbf{D}}(\mathbf{r},\tilde{\omega})e^{-i\tilde{\omega}t}. \tag{S13}$$

To calculate energy density of electromagnetic waves in dispersive media, we need to keep in mind the integration about angular frequency. This is especially true for QNMs which have a

finite linewidth in frequency. Then we have

$$\tilde{\mathbf{E}}^*(\mathbf{r},t)\cdot\tilde{\mathbf{D}}(\mathbf{r},t)=\int d\tilde{\omega}\int d\tilde{\omega}'\varepsilon(\tilde{\omega}')\tilde{\mathbf{E}}^*(\tilde{\omega})\cdot\tilde{\mathbf{E}}(\tilde{\omega}')e^{-i(\tilde{\omega}'-\tilde{\omega})t}. \tag{S14}$$

By assuming sufficiently narrow linewidth and taking first order approximation, we have $\tilde{\mathbf{E}}(\tilde{\omega})=\sum_n\tilde{\mathbf{E}}(\tilde{\omega}_n)\Delta(\tilde{\omega}-\tilde{\omega}_n)$ and $\tilde{\varepsilon}(\tilde{\omega})\approx\left.\frac{\partial(\tilde{\omega}\tilde{\varepsilon})}{\partial\tilde{\omega}}\right|_{\tilde{\omega}=\tilde{\omega}_0}$,respectively, where $\tilde{\mathbf{E}}(\tilde{\omega}_n)$ is n-th order QNM and $\Delta(\tilde{\omega}-\tilde{\omega}_n)$ is distribution function of n-th QNM in frequency domain. Then Eq. (S14) becomes

$$\tilde{\mathbf{E}}^*(\mathbf{r},t)\cdot\tilde{\mathbf{D}}(\mathbf{r},t)=\left.\frac{\partial(\tilde{\omega}\tilde{\varepsilon})}{\partial\tilde{\omega}}\right|_{\tilde{\omega}=\tilde{\omega}_0}\sum_{n,m}\int d\tilde{\omega}\int d\tilde{\omega}'\tilde{\mathbf{E}}^*(\tilde{\omega}_n)\Delta(\tilde{\omega}-\tilde{\omega}_n)\cdot\tilde{\mathbf{E}}(\tilde{\omega}_m)\Delta(\tilde{\omega}'-\tilde{\omega}_m)e^{-i(\tilde{\omega}'-\tilde{\omega})t} \tag{S15}$$

For a single QNM, $\tilde{\omega}_n=\tilde{\omega}_m=\tilde{\omega}_0$ and $\Delta(\tilde{\omega}-\tilde{\omega}_n)=\delta(\tilde{\omega}-\tilde{\omega}_0)$. Above derivation holds for $\tilde{\mathbf{H}}$ and $\tilde{\mathbf{B}}$. As a result, Eq. (S8) and (S9) in dispersive media become

$$\nabla\cdot\mathrm{Re}\left(\tilde{\mathbf{E}}\times\tilde{\mathbf{H}}^*\right)=-\mathrm{Im}\left[\frac{\partial(\tilde{\omega}\tilde{\varepsilon})}{\partial\tilde{\omega}}\right]_{\tilde{\omega}=\tilde{\omega}_0}\left|\tilde{\mathbf{E}}\right|^2-\mathrm{Im}\left[\frac{\partial(\tilde{\omega}\tilde{\mu})}{\partial\tilde{\omega}}\right]_{\tilde{\omega}=\tilde{\omega}_0}\left|\tilde{\mathbf{H}}\right|^2, \tag{S16}$$

$$\nabla\cdot\mathrm{Im}\left(\tilde{\mathbf{E}}\times\tilde{\mathbf{H}}^*\right)=-\mathrm{Re}\left[\frac{\partial(\tilde{\omega}\tilde{\varepsilon})}{\partial\tilde{\omega}}\right]_{\tilde{\omega}=\tilde{\omega}_0}\left|\tilde{\mathbf{E}}\right|^2+\mathrm{Re}\left[\frac{\partial(\tilde{\omega}\tilde{\mu})}{\partial\tilde{\omega}}\right]_{\tilde{\omega}=\tilde{\omega}_0}\left|\tilde{\mathbf{H}}\right|^2. \tag{S17}$$

4. Details in the 1D Fabry-Perot cavity

Here, we provide the details in the 1D Fabry-Perot cavity. The magnetic field inside the cavity $(0\le z\le d)$ can be obtained by

$$\tilde{\mathbf{H}}=\frac{1}{i\tilde{\omega}\mu_0}\nabla\times\mathbf{E}=\frac{\tilde{k}_2}{\tilde{\omega}\mu_0}\tilde{E}_2\left(\tilde{r}_{21}e^{i\tilde{k}_2z}-e^{-i\tilde{k}_2z}\right)e^{-i\tilde{\omega}_0t}\hat{\mathbf{y}}=\frac{n_2}{\eta_0}\tilde{E}_2\left(\tilde{r}_{21}e^{i\tilde{k}_2z}-e^{-i\tilde{k}_2z}\right)e^{-i\tilde{\omega}_0t}\hat{\mathbf{y}}, \tag{S18}$$

where we used $\tilde{k}_2=n_2\tilde{k}_0$, $\tilde{\omega}=c\tilde{k}_0$, and $c\mu_0=\eta_0$. The absolute squares of the electric and the magnetic fields are given by

$$\left|\tilde{\mathbf{E}}\right|^2=\left|\tilde{E}_2\right|^2\left\{\left|\tilde{r}_{21}\right|^2e^{-2\mathrm{Im}(\tilde{k}_2)z}+e^{+2\mathrm{Im}(\tilde{k}_2)z}+2\,\mathrm{Re}\left[\tilde{r}_{21}e^{2i\mathrm{Re}(\tilde{k}_2)z}\right]\right\}e^{-2\gamma_0t}, \tag{S19}$$

$$\left|\tilde{\mathbf{H}}\right|^2=\frac{\varepsilon_2}{\mu_0}\left|\tilde{E}_2\right|^2\left\{\left|\tilde{r}_{21}\right|^2e^{-2\mathrm{Im}(\tilde{k}_2)z}+e^{+2\mathrm{Im}(\tilde{k}_2)z}-2\,\mathrm{Re}\left[\tilde{r}_{21}e^{2i\mathrm{Re}(\tilde{k}_2)z}\right]\right\}e^{-2\gamma_0t}. \tag{S20}$$

From Eqs. (S19) and (S20), we obtain

$$\varepsilon_2\left|\tilde{\mathbf{E}}\right|^2+\mu_0\left|\tilde{\mathbf{H}}\right|^2=2\varepsilon_2\left|\tilde{E}_2\right|^2\left[\left|\tilde{r}_{21}\right|^2e^{-2\mathrm{Im}\left(\tilde{k}_2\right)z}+e^{+2\mathrm{Im}\left(\tilde{k}_2\right)z}\right]e^{-2\gamma_0 t}, \tag{S21}$$

$$\varepsilon_2\left|\tilde{\mathbf{E}}\right|^2-\mu_0\left|\tilde{\mathbf{H}}\right|^2=4\varepsilon_2\left|\tilde{E}_2\right|^2\mathrm{Re}\left[\tilde{r}_{21}e^{2i\mathrm{Re}\left(\tilde{k}_2\right)z}\right]e^{-2\gamma_0 t}. \tag{S22}$$

On the other hand, the cross product is given by

$$\tilde{\mathbf{E}}\times\tilde{\mathbf{H}}^*=\frac{n_2}{\eta_0}\left|\tilde{E}_2\right|^2\left\{\left|\tilde{r}_{21}\right|^2e^{-2\mathrm{Im}\left(\tilde{k}_2\right)z}-e^{+2\mathrm{Im}\left(\tilde{k}_2\right)z}-2i\,\mathrm{Im}\left[\tilde{r}_{21}e^{2i\mathrm{Re}\left(\tilde{k}_2\right)z}\right]\right\}e^{-2\gamma_0 t}\hat{\mathbf{z}}. \tag{S23}$$

We obtain the real and imaginary parts of the divergence of Eq. (S23),

$$\nabla\cdot\mathrm{Re}\left(\tilde{\mathbf{E}}\times\tilde{\mathbf{H}}^*\right)=-\frac{2n_2}{\eta_0}\left|\tilde{E}_2\right|^2\mathrm{Im}\left(\tilde{k}_2\right)\left[\left|\tilde{r}_{21}\right|^2e^{-2\mathrm{Im}\left(\tilde{k}_2\right)z}+e^{+2\mathrm{Im}\left(\tilde{k}_2\right)z}\right]e^{-2\gamma_0 t}, \tag{S24}$$

$$\nabla\cdot\mathrm{Im}\left(\tilde{\mathbf{E}}\times\tilde{\mathbf{H}}^*\right)=-\frac{4n_2}{\eta_0}\left|\tilde{E}_2\right|^2\mathrm{Re}\left(\tilde{k}_2\right)\mathrm{Re}\left[\tilde{r}_{21}e^{2i\mathrm{Re}\left(\tilde{k}_2\right)z}\right]e^{-2\gamma_0 t}. \tag{S25}$$

The ratio between the active (reactive) momentum and energy provides

$$\frac{\nabla\cdot\mathrm{Re}\left(\tilde{\mathbf{E}}\times\tilde{\mathbf{H}}^*\right)}{\varepsilon_2\left|\tilde{\mathbf{E}}\right|^2+\mu_0\left|\tilde{\mathbf{H}}\right|^2}=-\frac{c}{n_2}\mathrm{Im}\left(\tilde{k}_2\right)=-\mathrm{Im}\left(\tilde{\omega}_0\right)=\gamma_0, \tag{S26}$$

$$\frac{\nabla\cdot\mathrm{Im}\left(\tilde{\mathbf{E}}\times\tilde{\mathbf{H}}^*\right)}{\varepsilon_2\left|\tilde{\mathbf{E}}\right|^2-\mu_0\left|\tilde{\mathbf{H}}\right|^2}=-\frac{c}{n_2}\mathrm{Re}\left(\tilde{k}_2\right)=-\omega_0, \tag{S27}$$

where we used $\varepsilon_0\eta_0=1/c$ and $\tilde{\omega}=c\tilde{k}_0=\omega_0-i\gamma_0$.

We calculate the active/reactive pair of the energy and the momentum on the left side of the cavity $\left(z\le 0\right)$. The magnetic field is given by

$$\tilde{\mathbf{H}}=\frac{1}{i\tilde{\omega}\mu_0}\nabla\times\mathbf{E}=-\frac{n_1}{\eta_0}\tilde{E}_1e^{-i\left(\tilde{k}_1 z-\tilde{\omega}_0 t\right)}\hat{\mathbf{y}}. \tag{S28}$$

The absolute squares of the electric and the magnetic fields are given by

$$\left|\tilde{\mathbf{E}}\right|^2=\left|\tilde{E}_1\right|^2e^{2\mathrm{Im}\left(\tilde{k}_1\right)z}e^{-2\gamma_0 t}, \tag{S29}$$

$$\left|\tilde{\mathbf{H}}\right|^2=\frac{\varepsilon_1}{\mu_0}\left|\tilde{E}_1\right|^2e^{2\mathrm{Im}\left(\tilde{k}_2\right)z}e^{-2\gamma_0 t}. \tag{S30}$$

From Eqs. (S19) and (S20), we obtain

$$\varepsilon_1\left|\tilde{\mathbf{E}}\right|^2+\mu_0\left|\tilde{\mathbf{H}}\right|^2=2\varepsilon_1\left|\tilde{E}_1\right|^2e^{2\mathrm{Im}\left(\tilde{k}_1\right)z}e^{-2\gamma_0 t}, \tag{S31}$$

$$\varepsilon_1 \left|\tilde{\mathbf{E}}\right|^2 - \mu_0 \left|\tilde{\mathbf{H}}\right|^2 = 0 . \tag{S32}$$

On the other hand, the cross product is given by

$$\tilde{\mathbf{E}} \times \tilde{\mathbf{H}}^* = \frac{n_1}{\eta_0} \left|\tilde{E}_1\right|^2 e^{2\mathrm{Im}\left(\tilde{k}_1\right)z} e^{-2\gamma_0 t} \hat{\mathbf{z}} . \tag{S33}$$

We obtain the real and imaginary parts of the divergence of Eq. (S23),

$$\nabla \cdot \mathrm{Re}\left(\tilde{\mathbf{E}} \times \tilde{\mathbf{H}}^*\right) = \frac{2n_1}{\eta_0} \left|\tilde{E}_1\right|^2 \mathrm{Im}\left(\tilde{k}_1\right) e^{2\mathrm{Im}\left(\tilde{k}_1\right)z} e^{-2\gamma_0 t} , \tag{S34}$$

$$\nabla \cdot \mathrm{Im}\left(\tilde{\mathbf{E}} \times \tilde{\mathbf{H}}^*\right) = 0 . \tag{S35}$$

The ratio between momentum and energy is only valid for the active quantities.

$$\frac{\nabla \cdot \mathrm{Re}\left(\tilde{\mathbf{E}} \times \tilde{\mathbf{H}}^*\right)}{\varepsilon_1 \left|\tilde{\mathbf{E}}\right|^2 + \mu_0 \left|\tilde{\mathbf{H}}\right|^2} = -\frac{c}{n_1} \mathrm{Im}\left(\tilde{k}_1\right) = -\mathrm{Im}\left(\tilde{\omega}_0\right) = \gamma_0 \tag{S36}$$

Note that The ratio between the reactive momentum and the reactive energy is indeterminate.

5. Details in SPPs at the dielectric/metal interface

We revisit SPPs via QNM formalism. The p-polarized electromagnetic field in the *i*-th medium (*i*=1 and 2 for dielectric and metal, respectively) is given by

$$\tilde{\mathbf{E}}_i = \tilde{E}_{i,x} \begin{pmatrix} 1 \\ 0 \\ -\tilde{k}_x / \tilde{k}_{i,z} \end{pmatrix} e^{i\left(\tilde{k}_x x + \tilde{k}_{i,z} z - \tilde{\omega}_0 t\right)} , \tag{S37}$$

$$\begin{aligned} \tilde{\mathbf{H}}_i &= \frac{1}{i\tilde{\omega}\mu_0} \nabla \times \tilde{\mathbf{E}}_i \\ &= \frac{1}{c\mu_0} \tilde{E}_{i,x} \left(\frac{\tilde{k}_x^2 + \tilde{k}_{i,z}^2}{\tilde{k}_0 \tilde{k}_{i,z}} \right) e^{i\left(\tilde{k}_x x + \tilde{k}_{i,z} z - \tilde{\omega}_0 t\right)} \hat{\mathbf{y}} , \\ &= \frac{1}{\eta_0} \tilde{E}_{i,x} \left(\frac{\tilde{\varepsilon}_i \tilde{k}_0}{\varepsilon_0 \tilde{k}_{i,z}} \right) e^{i\left(\tilde{k}_x x + \tilde{k}_{i,z} z - \tilde{\omega}_0 t\right)} \hat{\mathbf{y}} \end{aligned} \tag{S38}$$

where we used the relation,

$$\left(\varepsilon_i / \varepsilon_0\right) \tilde{k}_0^2 = \tilde{k}_x^2 + \tilde{k}_{i,z}^2 . \tag{S39}$$

From Eq. (S37), the boundary conditions and the surface-bound solution condition impose

$$\varepsilon_1 \tilde{k}_{2,z} - \tilde{\varepsilon}_2 \tilde{k}_{1,z} = 0. \tag{S40}$$

Combining Eqs. (S39) and (S40), we obtain the famous relation for SPPs,

$$\tilde{k}_x^2 = \frac{1}{\varepsilon_0} \frac{\varepsilon_1 \tilde{\varepsilon}_2}{\varepsilon_1 + \tilde{\varepsilon}_2} \tilde{k}_0^2 = \frac{1}{\varepsilon_0} \frac{\varepsilon_1 \tilde{\varepsilon}_2}{\varepsilon_1 + \tilde{\varepsilon}_2} \frac{\tilde{\omega}_0^2}{c^2}, \tag{S41}$$

$$\tilde{k}_{1,z}^2 = \frac{1}{\varepsilon_0} \frac{\varepsilon_1^2}{\varepsilon_1 + \varepsilon_2} \tilde{k}_0^2, \tag{S42}$$

$$\tilde{k}_{2,z}^2 = \frac{1}{\varepsilon_0} \frac{\tilde{\varepsilon}_2^2}{\varepsilon_1 + \tilde{\varepsilon}_2} \tilde{k}_0^2. \tag{S43}$$

Since the expressions for the active/reactive energy and momentum become complicated in the dispersive medium 2 (i.e. metal), we calculate them in the medium 1 (i.e. dielectric). The absolute squares of the field are given by

$$\begin{aligned} \left|\tilde{\mathbf{E}}_1\right|^2 &= \left(\frac{\left|\tilde{k}_x\right|^2 + \left|\tilde{k}_{1,z}\right|^2}{\left|\tilde{k}_{i,z}\right|^2} \right) \left|\tilde{E}_{1,x}\right|^2 e^{-2\left[\mathrm{Im}\left(\tilde{k}_x\right)x + \mathrm{Im}\left(\tilde{k}_{j,z}\right)z\right]} e^{-2\gamma_0 t} \\ &= \frac{\varepsilon_1 \left|\tilde{k}_0\right|^2}{\varepsilon_0 \left|\tilde{k}_{i,z}\right|^2} \left|\tilde{E}_{1,x}\right|^2 e^{-2\left[\mathrm{Im}\left(\tilde{k}_x\right)x + \mathrm{Im}\left(\tilde{k}_{j,z}\right)z\right]} e^{-2\gamma_0 t} \end{aligned}, \tag{S44}$$

$$\left|\tilde{\mathbf{H}}_1\right|^2 = \frac{1}{\mu_0} \frac{\varepsilon_1^2 \left|\tilde{k}_0\right|^2}{\varepsilon_0 \left|\tilde{k}_{i,z}\right|^2} \left|\tilde{E}_{1,x}\right|^2 e^{-2\left[\mathrm{Im}\left(\tilde{k}_x\right)x + \mathrm{Im}\left(\tilde{k}_{j,z}\right)z\right]} e^{-2\gamma_0 t}, \tag{S45}$$

where we used the relation $\left(\varepsilon_1 / \varepsilon_0\right)\left|\tilde{k}_0\right|^2 = \left|\tilde{k}_x\right|^2 + \left|\tilde{k}_{1,z}\right|^2$. The active and reactive energy density are given by

$$\varepsilon_1 \left|\tilde{\mathbf{E}}_1\right|^2 + \mu_0 \left|\tilde{\mathbf{H}}_1\right|^2 = \frac{2\varepsilon_1^2 \left|\tilde{k}_0\right|^2}{\varepsilon_0 \left|\tilde{k}_{i,z}\right|^2} \left|\tilde{E}_{1,x}\right|^2 e^{-2\left[\mathrm{Im}\left(\tilde{k}_x\right)x + \mathrm{Im}\left(\tilde{k}_{j,z}\right)z\right]} e^{-2\gamma_0 t}, \tag{S46}$$

$$\varepsilon_1 \left|\tilde{\mathbf{E}}_1\right|^2 - \mu_0 \left|\tilde{\mathbf{H}}_1\right|^2 = 0. \tag{S47}$$

The cross product term is given by

$$\tilde{\mathbf{E}}_1 \times \tilde{\mathbf{H}}_1^* = \frac{\tilde{k}_0^*}{\eta_0} \frac{\varepsilon_1}{\varepsilon_0} \begin{pmatrix} \tilde{k}_x / \left|\tilde{k}_{1,z}\right|^2 \\ 0 \\ 1/\tilde{k}_{1,z}^* \end{pmatrix} \left|\tilde{E}_{1,x}\right|^2 e^{-2\left[\mathrm{Im}\left(\tilde{k}_x\right)x + \mathrm{Im}\left(\tilde{k}_{j,z}\right)z\right]} e^{-2\gamma_0 t}. \tag{S48}$$

Its divergence is written as

$$
\begin{aligned}
\nabla\cdot\left(\tilde{\mathbf{E}}_1\times\tilde{\mathbf{H}}_1^*\right) &= -2\frac{\tilde{k}_0^*}{\eta_0}\frac{\varepsilon_1}{\varepsilon_0}\left(\frac{\tilde{k}_x\,\mathrm{Im}\left(\tilde{k}_x\right)}{\left|\tilde{k}_{1,z}\right|^2}+\frac{\mathrm{Im}\left(\tilde{k}_{1,z}\right)}{\tilde{k}_{1,z}^*}\right)\left|\tilde{E}_{1,x}\right|^2 e^{-2\left[\mathrm{Im}\left(\tilde{k}_x\right)x+\mathrm{Im}\left(\tilde{k}_{j,z}\right)z\right]}e^{-2\gamma_0 t} \\
&= \frac{i\tilde{k}_0^*}{\eta_0}\frac{\varepsilon_1}{\varepsilon_0}\left[\frac{\tilde{k}_x\left(\tilde{k}_x-\tilde{k}_x^*\right)}{\left|\tilde{k}_{1,z}\right|^2}+\frac{\left(\tilde{k}_{1,z}-\tilde{k}_{1,z}^*\right)}{\tilde{k}_{1,z}^*}\right]\left|\tilde{E}_{1,x}\right|^2 e^{-2\left[\mathrm{Im}\left(\tilde{k}_x\right)x+\mathrm{Im}\left(\tilde{k}_{j,z}\right)z\right]}e^{-2\gamma_0 t} \\
&= \frac{i\tilde{k}_0^*}{\eta_0}\frac{\varepsilon_1}{\varepsilon_0}\left[\frac{\tilde{k}_x^2+\tilde{k}_{1,z}^2-\left(\left|\tilde{k}_x\right|^2+\left|\tilde{k}_{1,z}\right|^2\right)}{\left|\tilde{k}_{1,z}\right|^2}\right]\left|\tilde{E}_{1,x}\right|^2 e^{-2\left[\mathrm{Im}\left(\tilde{k}_x\right)x+\mathrm{Im}\left(\tilde{k}_{j,z}\right)z\right]}e^{-2\gamma_0 t} \\
&= -\frac{2}{\eta_0}\left(\frac{\varepsilon_1}{\varepsilon_0}\right)^2\frac{\mathrm{Im}\left(\tilde{k}_0\right)\left|\tilde{k}_0\right|^2}{\left|\tilde{k}_{1,z}\right|^2}\left|\tilde{E}_{1,x}\right|^2 e^{-2\left[\mathrm{Im}\left(\tilde{k}_x\right)x+\mathrm{Im}\left(\tilde{k}_{j,z}\right)z\right]}e^{-2\gamma_0 t}
\end{aligned}
\quad . \tag{S49}
$$

We can find (S49) has no imaginary part. Then, the ratio between active momentum and energy is given by

$$
\frac{\nabla\cdot\left(\tilde{\mathbf{E}}_1\times\tilde{\mathbf{H}}_1^*\right)}{\varepsilon_1\left|\tilde{\mathbf{E}}_1\right|^2+\mu_0\left|\tilde{\mathbf{H}}_1\right|^2} = -\frac{1}{\varepsilon_0\eta_0}\mathrm{Im}\left(\tilde{k}_0\right) = -c\,\mathrm{Im}\left(\tilde{k}_0\right) = -\mathrm{Im}\left(\tilde{\omega}_0\right) = \gamma_0, \tag{S50}
$$

while the ratio between reactive momentum and energy is indeterminate.

6. Conservation law of the active/reactive optical helicity and the spin angular momentum of light

Spin angular momentum density **s** is defined by the momentum density **p** via the total angular momentum density $\mathbf{j}=\mathbf{r}\times\mathbf{p}$ with the position vector **r**. Then, the angular momentum $\int \mathbf{j}dV$ can be decomposed into $\int \mathbf{s}dV+\int\left(\mathbf{r}\times\mathbf{p}_L\right)dV$ where s has no reference to r, while the orbital angular momentum density is defined by $\mathbf{l}\equiv\mathbf{r}\times\mathbf{p}_L$ with the orbital part of the momentum $\mathbf{p}_L$ [2], [3]. Here, we summarize the result of the decomposition of the momentum. To write the spin angular momentum density and the helicity in a simple form, one can define the gauge-dependent electric vector potential **C**, using the relation $\mathbf{D}=-\nabla\times\mathbf{C}$ [4]. The helicity and spin angular momentum densities are defined by [4]

$$
h=\frac{1}{2}\left\{\sqrt{\frac{\varepsilon_0}{\mu_0}}\mathbf{A}\cdot\left(\nabla\times\mathbf{A}\right)+\sqrt{\frac{\mu_0}{\varepsilon_0}}\mathbf{C}\cdot\left(\nabla\times\mathbf{C}\right)\right\}, \tag{S51}
$$

$$
\mathbf{s}=\frac{1}{2}\left(\varepsilon_0\mathbf{E}\times\mathbf{A}+\mu_0\mathbf{H}\times\mathbf{C}\right), \tag{S52}
$$

where **A** is the (magnetic) vector potential defined by $\mathbf{B}=\nabla\times\mathbf{A}$. Two quantities satisfy the continuity equation,

$$\frac{\partial h}{\partial t} + c\nabla \cdot \mathbf{s} = 0. \tag{S53}$$

For the harmonic field with the real frequency, the conserved quantities mentioned above are written in the forms,

$$h = \frac{1}{4}\mathrm{Re}\left\{\sqrt{\frac{\varepsilon_0}{\mu_0}}\mathbf{A}^* \cdot (\nabla \times \mathbf{A}) + \sqrt{\frac{\mu_0}{\varepsilon_0}}\mathbf{C}^* \cdot (\nabla \times \mathbf{C})\right\}, \tag{S54}$$

$$\mathbf{s} = \frac{1}{4}\mathrm{Re}\left(\varepsilon_0 \mathbf{E}^* \times \mathbf{A} + \mu_0 \mathbf{H}^* \times \mathbf{C}\right), \tag{S55}$$

while the continuity equation can also be written as

$$-i\omega h + c\nabla \cdot \mathbf{s} = 0. \tag{S56}$$

In addition, Eqs. (S54) and (S55) can be written in terms of $\{\tilde{\mathbf{E}}, \tilde{\mathbf{H}}\}$. If we choose vector potentials satisfying $\mathbf{E} = -\partial \mathbf{A}/\partial t$ and $\mathbf{H} = -\partial \mathbf{C}/\partial t$, i.e. $\tilde{\mathbf{E}} = i\omega \tilde{\mathbf{A}}$ and $\tilde{\mathbf{H}} = i\omega \tilde{\mathbf{C}}$, we obtain

$$h = \frac{1}{2\omega c}\mathrm{Im}\left(\tilde{\mathbf{E}} \cdot \tilde{\mathbf{H}}^*\right), \tag{S57}$$

$$\mathbf{s} = -\frac{1}{4\omega}\mathrm{Im}\left(\varepsilon_0 \tilde{\mathbf{E}} \times \tilde{\mathbf{E}}^* + \mu_0 \tilde{\mathbf{H}} \times \tilde{\mathbf{H}}^*\right). \tag{S58}$$

For the time-harmonic circularly polarized plane waves, we can find from Eq. (S57) that the ratio becomes $h/u = \pm 1/\omega$ where the energy density of the plane wave is given by $u = \varepsilon_0 \left|\tilde{\mathbf{E}}\right|^2 / 2$. The continuity equation provides a physical interpretation of the helicity, *i.e.* $h = \hat{\mathbf{k}} \cdot \mathbf{s}$ where $\hat{\mathbf{k}}$ denotes the direction of the wavevector **k** of the plane wave.

Here, we perform the same calculation for the electromagnetic field of the quasinormal modes. Following the standard derivation of the spin and orbital angular momentum from the momentum [2], [3], we decompose the complex momentum density $\tilde{\mathbf{p}}$ into the spin and orbital parts, i.e. $\tilde{\mathbf{p}} = \tilde{\mathbf{p}}_L + \tilde{\mathbf{p}}_S$:

$$\tilde{\mathbf{p}}_S = -\frac{1}{4}\left\{\varepsilon_0\left(\tilde{\mathbf{E}} \cdot \nabla\right)\tilde{\mathbf{A}}^* + \mu_0\left(\tilde{\mathbf{H}}^* \cdot \nabla\right)\tilde{\mathbf{C}}\right\}, \tag{S59}$$

$$\tilde{\mathbf{p}}_L = \frac{1}{4}\left\{\varepsilon_0 \tilde{\mathbf{E}} \cdot \nabla \tilde{\mathbf{A}}^* + \mu_0 \tilde{\mathbf{H}}^* \cdot \nabla \tilde{\mathbf{C}}\right\}. \tag{S60}$$

Here we have the relations for the QNM fields: $\tilde{\mathbf{E}} = i\tilde{\omega}_0 \tilde{\mathbf{A}}$, $\tilde{\mathbf{H}} = i\tilde{\omega}_0 \tilde{\mathbf{C}}$, and $\tilde{\mathbf{D}} = -\nabla \times \tilde{\mathbf{C}}$. We calculate the complex spin angular momentum by the integration,

$$\begin{aligned}\int \tilde{\mathbf{s}} dV &= \int \left(\mathbf{r}\times\tilde{\mathbf{p}}_S\right) dV \\ &= -\frac{1}{4}\int\left\{\varepsilon_0 \mathbf{r}\times\left(\tilde{\mathbf{E}}\cdot\nabla\right)\tilde{\mathbf{A}}^* + \mu_0 \mathbf{r}\times\left(\tilde{\mathbf{H}}^*\cdot\nabla\right)\tilde{\mathbf{C}}\right\} dV \\ &= \frac{1}{4}\int\left(\varepsilon_0\tilde{\mathbf{E}}\times\tilde{\mathbf{A}}^* + \mu_0\tilde{\mathbf{H}}^*\times\tilde{\mathbf{C}}\right) dV\end{aligned} \tag{S61}$$

Therefore, we can define the complex spin angular momentum density $\tilde{\mathbf{s}}$ in the form,

$$\tilde{\mathbf{s}} \equiv \frac{1}{4}\left(\varepsilon_0\tilde{\mathbf{E}}\times\tilde{\mathbf{A}}^* + \mu_0\tilde{\mathbf{H}}^*\times\tilde{\mathbf{C}}\right). \tag{S62}$$

Here, we define the spin angular momentum density $\mathbf{s}\equiv\mathrm{Re}\left(\tilde{\mathbf{s}}\right)$ and reactive spin angular momentum density $\overline{\mathbf{s}}=\mathrm{Im}\left(\tilde{\mathbf{s}}\right)$ as the real and imaginary part of the complex spin angular momentum density, *i.e.* $\tilde{\mathbf{s}}=\mathbf{s}+i\overline{\mathbf{s}}$. Note that $\mathbf{s}\equiv\mathrm{Re}\left(\tilde{\mathbf{s}}\right)$ for the QNM field has the same form of Eq. (S55) for the ordinary harmonic fields.

To derive the continuity equation for the complex spin angular momentum density $\tilde{\mathbf{s}}$, we take the divergence of Eq. (S62), yielding

$$\nabla\cdot\tilde{\mathbf{s}} = -\frac{1}{2c^2}\left\{\tilde{\omega}_0\,\mathrm{Im}\left(\tilde{\mathbf{A}}^*\cdot\tilde{\mathbf{H}}\right)+\tilde{\omega}_0^*\,\mathrm{Im}\left(\tilde{\mathbf{C}}\cdot\tilde{\mathbf{E}}^*\right)\right\}. \tag{S63}$$

The real part of Eq. (S63) gives

$$c\,\mathrm{Re}\left(\nabla\cdot\tilde{\mathbf{s}}\right) = -\frac{1}{2c}\omega_0\left\{\mathrm{Im}\left(\tilde{\mathbf{A}}^*\cdot\tilde{\mathbf{H}}\right)+\mathrm{Im}\left(\tilde{\mathbf{C}}\cdot\tilde{\mathbf{E}}^*\right)\right\}. \tag{S64}$$

Imposing Eq. (S64) to be the form of the continuity equation, $c\nabla\cdot\mathbf{s}/2+\mathrm{Re}\left(-i\tilde{\omega}_0\right)h=0$, we can define the helicity density *h* in the form,

$$h = -\frac{1}{4c}\frac{\omega_0}{\gamma_0}\left\{\mathrm{Im}\left(\tilde{\mathbf{A}}^*\cdot\tilde{\mathbf{H}}\right)+\mathrm{Im}\left(\tilde{\mathbf{C}}\cdot\tilde{\mathbf{E}}^*\right)\right\} \tag{S65}$$

The imaginary part of Eq. (S63) gives

$$c\,\mathrm{Im}\left(\nabla\cdot\tilde{\mathbf{s}}\right) = \frac{1}{2c}\gamma_0\left\{\mathrm{Im}\left(\tilde{\mathbf{A}}^*\cdot\tilde{\mathbf{H}}\right)-\mathrm{Im}\left(\tilde{\mathbf{C}}\cdot\tilde{\mathbf{E}}^*\right)\right\} \tag{S66}$$

Likewise, by $c\nabla\cdot\overline{\mathbf{s}}/2+\mathrm{Im}\left(-i\tilde{\omega}_0\right)\overline{h}=0$, we can define the reactive helicity density $\overline{h}$ in the form,

$$\overline{h} = \frac{1}{4c}\frac{\gamma_0}{\omega_0}\left\{\mathrm{Im}\left(\tilde{\mathbf{A}}^*\cdot\tilde{\mathbf{H}}\right)-\mathrm{Im}\left(\tilde{\mathbf{C}}\cdot\tilde{\mathbf{E}}^*\right)\right\} \tag{S67}$$

We can write Eqs. (S62), (S65), and (S67) in terms of $\left\{\tilde{\mathbf{E}},\tilde{\mathbf{H}}\right\}$ as follows:

$$\tilde{\mathbf{s}} = -\frac{1}{4i}\left(\frac{\varepsilon_0}{\tilde{\omega}_0^*}\tilde{\mathbf{E}}\times\tilde{\mathbf{E}}^* + \frac{\mu_0}{\tilde{\omega}_0}\tilde{\mathbf{H}}\times\tilde{\mathbf{H}}^*\right), \tag{S68}$$

$$h = -\frac{1}{2c}\frac{\omega_0}{|\tilde{\omega}_0|^2}\mathrm{Im}\left(\tilde{\mathbf{E}}^*\cdot\tilde{\mathbf{H}}\right), \tag{S69}$$

$$\bar{h} = \frac{1}{2c}\frac{\gamma_0}{|\tilde{\omega}_0|^2}\mathrm{Re}\left(\tilde{\mathbf{E}}\cdot\tilde{\mathbf{H}}^*\right). \tag{S70}$$

Again, converting the two QNM continuity equations, $c\nabla\cdot\mathbf{s}/2+\mathrm{Re}\left(-i\tilde{\omega}_0\right)h=0$ and $c\nabla\cdot\bar{\mathbf{s}}/2+\mathrm{Im}\left(-i\tilde{\omega}_0\right)\bar{h}=0$ into the integral forms, we can obtain Eqs. (7) and (8) in the main text. Note that the spin and reactive spin angular momenta are explicitly given by

$$\mathbf{s} = \mathrm{Re}\left(\tilde{\mathbf{s}}\right) = -\frac{1}{4}\mathrm{Im}\left(\frac{\varepsilon_0}{\tilde{\omega}_0^*}\tilde{\mathbf{E}}\times\tilde{\mathbf{E}}^* + \frac{\mu_0}{\tilde{\omega}_0}\tilde{\mathbf{H}}\times\tilde{\mathbf{H}}^*\right), \tag{S71}$$

$$\bar{\mathbf{s}} = \mathrm{Im}\left(\tilde{\mathbf{s}}\right) = \frac{1}{4}\mathrm{Re}\left(\frac{\varepsilon_0}{\tilde{\omega}_0^*}\tilde{\mathbf{E}}\times\tilde{\mathbf{E}}^* + \frac{\mu_0}{\tilde{\omega}_0}\tilde{\mathbf{H}}\times\tilde{\mathbf{H}}^*\right). \tag{S72}$$

References

[1] J. D. Jackson, *Classical Electrodynamics*, 3rd ed., vol. 3. New York: Wiley, 1999.

[2] R. P. Cameron, S. M. Barnett, and A. M. Yao, “Optical helicity, optical spin and related quantities in electromagnetic theory,” *New J Phys*, vol. 14, no. 5, p. 053050, May 2012, doi: 10.1088/1367-2630/14/5/053050.

[3] M. V Berry, “Optical currents,” *Journal of Optics A: Pure and Applied Optics*, vol. 11, no. 9, p. 094001, 2009, doi: 10.1088/1464-4258/11/9/094001.

[4] F. Crimin, N. Mackinnon, J. Götte, and S. Barnett, “Optical Helicity and Chirality: Conservation and Sources,” *Applied Sciences*, vol. 9, no. 5, p. 828, Feb. 2019, doi: 10.3390/app9050828.
